# Long-term clinical results and MRI changes after autologous chondrocyte implantation in the knee of young and active middle aged patients

**DOI:** 10.1007/s10195-015-0383-6

**Published:** 2015-10-24

**Authors:** Donato Rosa, Giovanni Balato, Giovanni Ciaramella, Ernesto Soscia, Giovanni Improta, Maria Triassi

**Affiliations:** Department of Public Health, School of Medicine, Federico II University, Via S. Pansini 5, Bl. 12, 80131 Naples, Italy; Institute of Biostructure and Bioimaging, National Research Council, Via S. Pansini 5, 80131 Naples, Italy

**Keywords:** Autologous chondrocyte implantation, Chondral lesion, Magnetic resonance imaging, Knee, Osteochondritis dissecans

## Abstract

**Background:**

Autologous chondrocyte implantation (ACI) represents a valid surgical option for symptomatic full-thickness chondral lesions of the knee. Here we report long-term clinical and MRI results of first-generation ACI.

**Materials and methods:**

Fifteen patients (mean age 21.3 years) underwent first-generation ACI for symptomatic chondral defects of the knee between 1997 and 2001. The mean size of the lesions was 5.08 cm^2^ (range 2–9 cm^2^). Patients were evaluated using the International Knee Documentation Committee (IKDC) Knee Examination Form, the Tegner Activity Scale, and the Knee Injury and Osteoarthritis Outcome Score (KOOS). High-resolution MRI was used to analyze the repair tissue with nine variables (the MOCART scoring system).

**Results:**

The mean follow-up period was 148 months (range 125–177 months). ACI resulted in substantial improvements in all clinical outcome parameters, even as much as 12 years after implantation. A significant decrease in the MOCART score was recorded at final measurement. Reoperation was required in 2 patients; failure was caused by partial detachment of the graft in both cases.

**Conclusion:**

Autologous chondrocyte implantation is an effective and durable solution for the treatment of large, full-thickness cartilage and osteochondral lesions, even in young and active middle-aged patients. High-resolution MRI is a useful and noninvasive method for evaluating the repaired tissue.

**Level of evidence:**

IV.

## Introduction

Cartilage lesions of the knee in orthopedic patients are an underestimated problem. Despite the advances made in scientific knowledge and technology, treatment of these lesions remains troublesome. Autologous chondrocyte implantation (ACI), first reported in 1994 by Brittberg et al., was introduced as an alternative means of treating symptomatic full-thickness chondral lesions of the knee [1]. After ACI, cartilage repair tissue consists mainly of cartilage-like tissue that mimics the macroscopic, microscopic, and biomechanical features of healthy hyaline cartilage [2, 3]. Magnetic resonance imaging (MRI) is the most reproducible and least aggressive technique for assessing cartilage regeneration after ACI. One validated scoring system for the morphologic MRI evaluation of cartilage repair sites is the Magnetic Resonance Observation of Cartilage Repair Tissue (MOCART) system [4, 5]. Although satisfactory results in mid-term pain relief have been reported [6], only a limited number of studies have examined the long-term results of ACI in terms of clinical assessment, patient satisfaction, and magnetic resonance imaging (MRI) results [7–13].

The purposes of the study reported in the present paper were therefore to:

- Evaluate the overall long-term results of ACI in terms of clinical assessment, patient satisfaction, and magnetic resonance imaging (MRI) results
- Compare the long-term with the short-term clinical results
- Evaluate the correlation between the subjective clinical outcome and the radiological MOCART scoring system and its variables.

## Materials and methods

Between 1997 and 2001, 15 patients (nine men and six women), with a mean age of 21 years (range 13–45), underwent autologous chondrocyte implantation using the original periosteum-cover technique. All patients had knee pain and had decreased their physical activity due to the presence of a chondral defect in the concerned knee. A symptomatic full-thickness cartilage lesion (Outerbridge grade III or IV) or an osteochondral lesion (2–12 cm^2^) was considered an indication for ACI. Exclusion criteria were age >45 years, prolonged osteoarthritis (Kellgren–Lawrence grade 2 or more), obesity (BMI > 35 kg/m^2^), a kissing lesion, active inflammatory arthritis or infection, varus/valgus alignment >5°, and/or untreated knee instability. No patient had undergone any previous surgical attempts to treat the chondral defect, except for one case in which a meniscectomy was performed together with a chondral debridement for a patellar lesion. A trauma was the cause of the chondral defect in eight cases, whilst osteochondritis dissecans was the underlying cause in seven cases. Patients with osteochondritis dissecans were rated International Cartilage Repair Society (ICRS) stage 2 (partial discontinuity, stable on probing) or 3 (having an unstable but not dislocated fragment). The mean size of the lesion surface was 5.08 ± 2.01 cm^2^ (range 2–9 cm^2^). Lesions were localized on the medial femoral condyle in 10 cases, on the lateral femoral condyle in two cases, on the patella in two cases, and on the tibial plateau in one case. During the first arthroscopic step, three partial meniscectomies (two medial and one lateral) were performed, while an anterior cruciate ligament (ACL) reconstruction and a patellar alignment (Tables 1, 2) were performed during the implantation of chondrocytes. All individuals provided oral and written informed consent for the publication of their individual clinical details in this paper; this was approved by the institutional review board of our department and is compliant with the Declaration of Helsinki.

**Table 1:** Patient demographic characteristics as well as prior and concomitant procedures

Patient characteristic	*N* = 15
Age, years	
Mean ± SD	21,33 ± 8,92
Range	13–45
Gender	
Male	9/15
Female	6/15
Previous procedures	
Debridement/lavage	1/15
Procedures performed concurrently with cartilage harvest	
Medial meniscectomy	2/15
Lateral meniscectomy	1/15
Procedures performed with implantation	
ACL reconstruction	1/15
Patellar alignment	1/15

*SD* standard deviation

**Table 2:** Characteristics of chondral lesions

Defect characteristic	
Acute traumatic injury	8/15
Osteochondritis dissecans	7/15
Total surface area, cm^2^	
Mean ± SD	5.08 ± 2.01
Range	2–9
Defect location	
Medial femoral condyle	10/15
Lateral femoral condyle	2/15
Patella	2/15
Tibial plate	1/15

*SD* standard deviation

### Surgical technique

The ACI technique consisted of a two-step procedure, as originally described by Brittberg [1]. First, an arthroscopy was performed, where small pieces of full-thickness cartilage were harvested from a low-weight-bearing area of the trochlea or from the upper area of the medial condyle; these pieces weighed approximately 200–300 mg. The biopsy material was placed in a nutrient medium and transported within 24 h to a chosen laboratory. Chondrocytes were isolated from the cartilage by enzymatic treatment, and the number of chondrocytes was increased via monolayer culture, as described previously [1]. After 3–4 weeks, an autologous pool of chondrocytes was ready to be implanted. The surgical approach used in the implantation step of the procedure depended on the size and location of the defect: a medial or lateral parapatellar arthrotomy was performed. Defect edges were marked and then cut using a surgical blade, creating a contained lesion with surrounding healthy cartilage. In all cases with osteochondritis dissecans, the lesion was identified and the fragment was removed along with fibrous tissue and degenerated bone until healthy, bleeding bone was reached. A periosteal flap was harvested from the proximal medial subcutaneous border of the tibia. An incision was made about 3 cm below the insertion of the pes anserinus. With the inner cambium layer facing the lesion, the periosteal flap was sutured to the surrounding cartilage using interrupted absorbable sutures. The periosteal rim was sealed with fibrin glue except for one corner, where the suspension of cultured chondrocytes (Carticel) was injected into the defect. The implant was completed by closing the corner with a final suture and the fibrin glue.

### Postoperative rehabilitation protocol

The goal of rehabilitation was to protect the graft while promoting maturation of the newly implanted chondrocytes by implementing a program that focused on regaining full range of motion (ROM), progressive weight bearing, lower extremity strengthening, flexibility, and proprioceptive training. In particular, when at least 24 h had passed following surgery, the knee was mobilized with the help of a continuous passive motion (CPM) machine. Weight-bearing activity was typically barred until after the first 2 weeks of implantation in order to preserve the physical properties of the graft. Partial weight bearing was then permitted until 4 weeks after surgery. From 4 to 6 weeks after surgery, the patient could progress to the use of one crutch, with the load gradually increased over the subsequent 6 weeks so that full weight bearing had occurred by week 12. By 3 months after surgery, the patient had recovered their full active range of motion with a normalized gait pattern. At 6–9 months after surgery, the patient continued progressive strength training and transitioned to more functional activities. From 9 to 18 months after surgery, the goal of the rehabilitation was to implement sports-specific activity and eventually facilitate the return of the patient to competition.

### Clinical evaluation

The International Knee Documentation Committee (IKDC) Knee Examination Form [14, 15] and the Tegner Activity Level Score [16] were used to perform clinical and functional evaluation at baseline. Before the surgical procedure/surgery, all of the patients underwent a physical examination, and weight-bearing standing radiographs as well as magnetic resonance images (MRIs) of the affected knees were recorded. Follow-up was accomplished in all patients for a mean period of 148.1 ± 15.76 months (range 125–177 months). Each year, a clinical evaluation was performed. At the final follow-up, functional evaluation was performed with the IKDC Knee Examination Form, the Tegner Activity Level Score, and the Knee Injury and Osteoarthritis Outcome Score (KOOS) (Italian version LK 1.0) [17, 18]. Cases in which further surgery was performed after ACI were defined as treatment failures.

### Radiological evaluation

Thirteen of the patients periodically underwent magnetic resonance imaging (1.5 T, Siemens Symphony) according to the following acquisition protocol:

- Axial TSE PD FS 2D 1-7/180 (TE 37, TR 2500, matrix 192 × 256, FOV 180 × 180).
- Sagittal TSE PD FS 2D 1-7/180 (TE 38, TR 2000, matrix 240 × 320, FOV 180 × 180).
- Coronal TSE PD FS 2D 1-7/180 (TE 37, TR 2000, matrix 256 × 256, FOV 180 × 180).
- Sagittal TSE PD 2D 1-7/180 (TE 38, TR 2000, matrix 240 × 320, FOV 180 × 180).
- Sagittal 3D spoiled GRE T1 (Fi 3D 1/40) (TE 8, TR 34, matrix 192 × 256, FOV 180 × 180).

The mean time of the first MRI after the implantation was 12 months (range 6–30 months). At final follow-up after a mean of 148.1 months (range 122–175 months), 11 patients were studied using a high-field MRI instrument (3 T, Siemens Magnetom Trio) that was available at the time in the radiologic department of our institution. The examination was performed with a dedicated knee coil. The acquisition protocol was as follows:

- Axial TSE PD FS (TE 11, TR5890, matrix 256 × 256, thickness 3 mm, FOV 160 × 160).
- Sagittal TSE PD FS (TE 11, TR4660, matrix 320 × 320, thickness 3 mm, FOV 160 × 160).
- Coronal FFE 3D T1 hi-res VIBE (Te5, Tr14,2, FA 25, slice thickness 0.6, matrix 512 × 512, FOV 150 × 150).
- Sagittal FFE DP 3D hi-res (Te232, Tr2200, matrix 230 × 250, FA 120, slice thickness 0.8, FOV 162 × 181).
- Sagittal T2 3D hi-res (Te4.9, Tr11, matrix 480 × 512, slice thickness 0.6, FA 40, FOV 140 × 150).

The images were evaluated by an expert radiologist according to the MOCART scoring system.

### Statistical analysis

Paired sample* t*-tests were used to determine whether follow-up data were significantly increased or decreased from the baseline clinical scores. In this context, the MRI score after short-term follow-up was compared with the long-term follow-up MOCART score by paired *t*-test. To determine the correlation between clinical outcome and MRI score, the KOOS and the IKDC scores were correlated with the MOCART score and with the nine variables of the MRI scoring system. For the statistical analysis, Spearman’s correlation coefficient (*r*_s_) and Student’s* t*-test were calculated. To evaluate the relationships of the MOCART score and some of its variables (degree of defect filling, integration of border zone, surface of the repair tissue, structure of the repair tissue, signal intensity of the repair tissue) with the KOOS variables and those of the IKDC, Spearman’s correlation coefficient was calculated considering the ranks of the variables, not their numerical values. An independent samples* t*-test was used for the remaining MOCART variables (subchondral lamina, subchondral bone, adhesions, effusion). All tests were performed using the statistical software package R (R Development Core Team, 2005). In all instances, *P* < 0.05 was regarded as statistically significant.

## Results

**Fig. 1:**
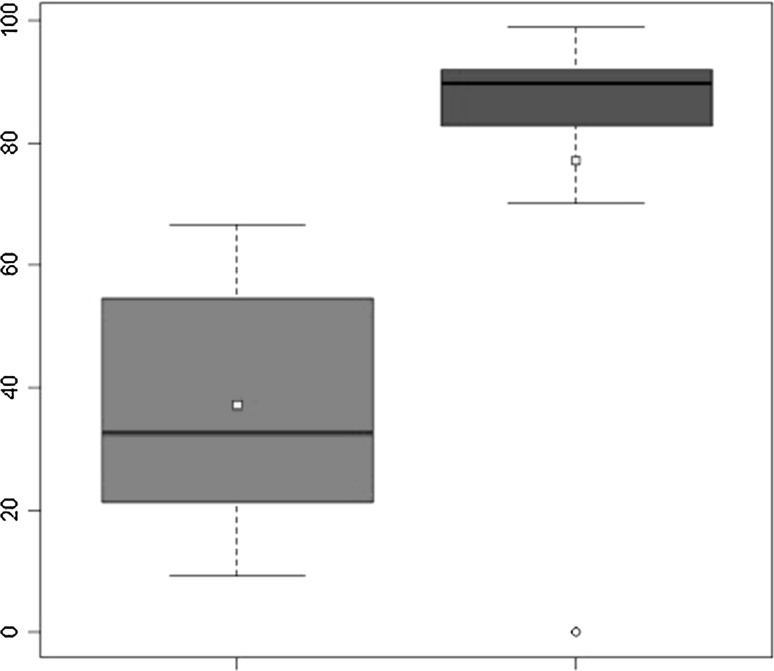
IKDC score: improvement from pre-operative levels to final follow-up

**Fig. 2:**
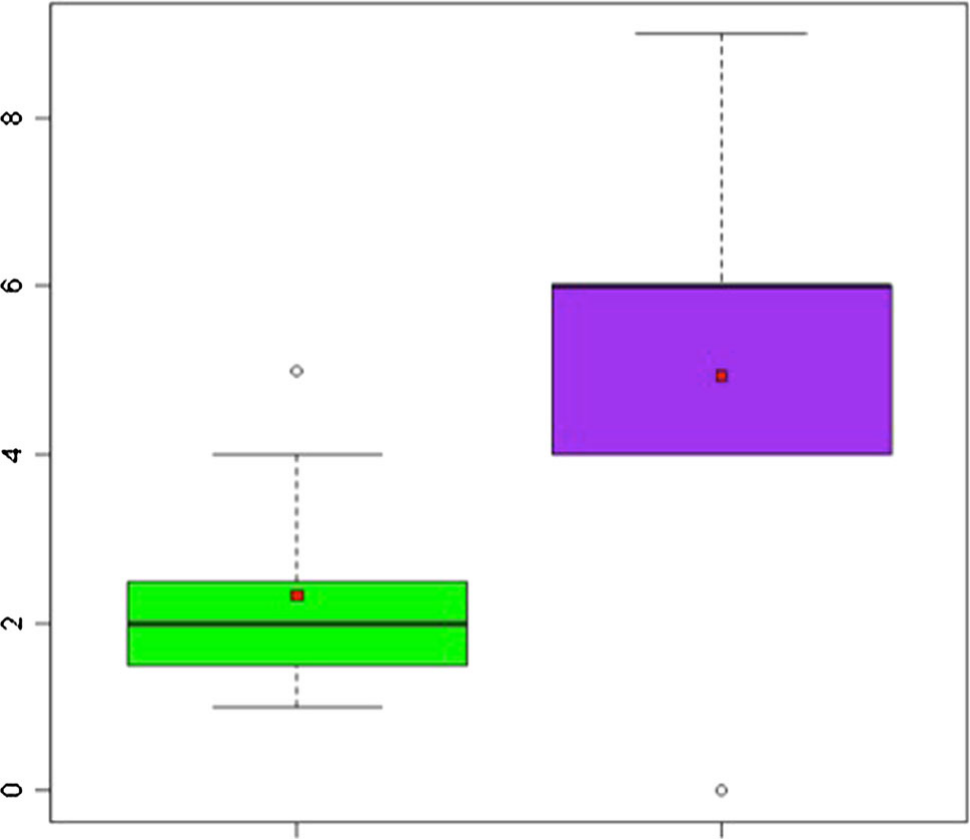
Tegner score: improvement from pre-operative levels to final follow-up

**Fig. 3:**
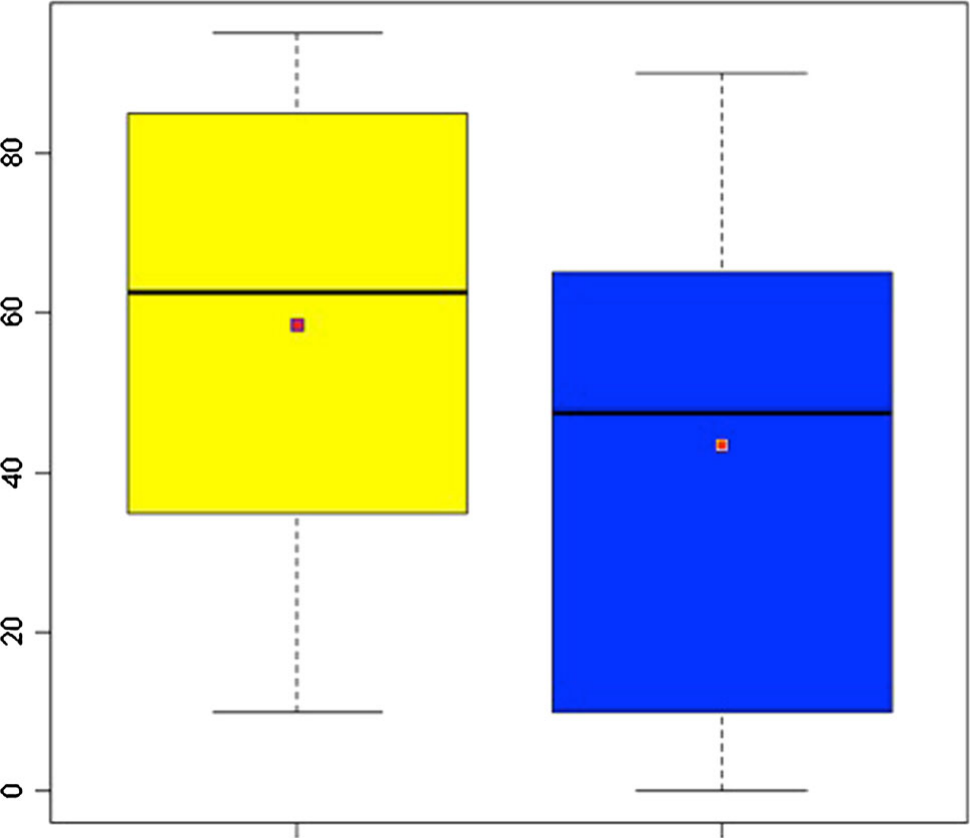
MOCART score: comparison of MOCART score at 12 months with that at final follow-up

All 15 patients were retrospectively followed up after ACI for a mean period of 148.1 ± 15.76 months (range 125–177 months). Two patients (13.3 %) needed an operation after ACI, entailing removal of the graft and treatment of the defect with microfractures. Failure was in both cases due to partial detachment of the graft and degeneration of the graft area. The graft site was also filled with fibrous tissue that was partially lifted at its medial aspect, exposing the subchondral bone. The defect area was debrided from the fibrous tissue and the chondral lesion was exposed. The microfracture technique was then performed as a treatment for the lesion. At the final follow-up, significant increases in all scores were recorded. Compared with the pre-procedure findings, the mean IKDC score improved significantly, increasing from 37.20 ± 19.54 to 76.32 ± 32.36 (*P* = 0.000314) (Fig. 1). The Tegner Activity Level Score showed significant improvement after surgery, increasing from 2.33 ± 1.34 to 4.93 ± 2.43 (*P* = 0.0011) (Fig. 2). The KOOS scores were as follows: pain 79.63 ± 33.33; symptoms 76.42 ± 32.47; ADL 85.09 ± 34.62; sport 70.33 ± 31.13; knee-related quality of life 74.17 ± 32.72. The mean MOCART score at the first follow-up was 55 ± 26.53, whereas that at the last follow-up was 45 ± 31.62. Ten patients underwent MRI at both short-term and long-term follow-ups; the paired *t*-test showed a significant decrease in the MOCART score from 59 ± 29.13 to 43.5 ± 32.91 (*P* = 0.0226) (Fig. 3). Table 3 shows the results for each variable of the MOCART score at both short-term and long-term follow-ups. The correlation coefficients and the results of the* t*-test for subjective outcomes and the different variables of the MRI classification system indicated that there were statistically significant correlations between degree of defect repair and pain KOOS as well as between effusion and the pain and symptoms KOOS (*p* < 0.05).

**Table 3:** MRI evaluation of repair tissue 1–2 years and 10 years after ACI implantation

Variables	First follow-up	Last follow-up
1. Degree of defect repair and filling of the defect		
Complete	2/10 (20 %)	2/10 (20 %)
Hypertrophy	4/10 (40 %)	4/10 (40 %)
Incomplete		
>50 % of the adjacent cartilage	3/10 (30 %)	0/10 (0 %)
<50 % of the adjacent cartilage	0/10 (0 %)	1/10 (10 %)
Subchondral bone exposed	1/10 (10 %)	3/10 (30 %)
2. Integration to border zone		
Complete	7/10 (70 %)	4/10 (40 %)
Incomplete		
Demarcating border visible (split-like)	0/10 (0 %)	1/10 (10 %)
Defect visible		
<50 % of the length of the repair tissue	1/10 (10 %)	2/10 (20 %)
>50 % of the length of the repair tissue	2/10 (20 %)	3/10 (30 %)
3. Surface of the repair tissue		
Surface intact	5/10 (50 %)	5/10 (50 %)
Surface damaged		
<50 % of repair tissue depth	2/10 (20 %)	2/10 (20 %)
>50 % of repair tissue depth or total degeneration	3/10 (30 %)	3/10 (30 %)
4. Structure of the repair tissue		
Homogeneous	3/10 (30 %)	1/10 (10 %)
Inhomogeneous or cleft formation	7/10 (70 %)	9/10 (90 %)
5. Signal intensity of the repair tissue		
Normal (identical to adjacent cartilage)	3/10 (30 %)	2/10 (20 %)
Nearly normal	5/10 (50 %)	5/10 (50 %)
Abnormal	2/10 (20 %)	3/10 (30 %)
6. Subchondral lamina		
Intact	2/10 20 %)	1/10 (10 %)
Not intact	8/10 (80 %)	9/10 (90 %)
7. Subchondral bone		
Intact	4/10 (40 %)	1/10 (10 %)
Not intact	6/10 (60 %)	9/10 (90 %)
8. Adhesions		
No	8/10 (80 %)	2/10 (20 %)
Yes	2/10 (20 %)	8/10 (80 %)
9. Effusions		
No	8/10 (80 %)	6/10 (60 %)
Yes	2/10 (20 %)	4/10 (40 %)

## Discussion

In this study, we evaluated the durability of ACI in patients treated for full-thickness cartilage defects. The most important finding of our study was confirmation of the long-term effectiveness of ACI, even up to 14 years after the first implantation performed in our center. Clinical and functional improvements, with significantly increased mean IKDC and Tegner Activity Level scores, were observed in 86.6 % of cases. A functional evaluation was also performed using the Knee Injury and Osteoarthritis Outcome Score (KOOS) at final follow-up. The KOOS results were compared with the age-specific KOOS scores for the general population, as obtained in the epidemiological study of Paradowski et al. [19]. The mean KOOS scores for the 18–34 year-old age group were 92.2 men/92.1 women for pain, 87.2 men/89.1 women for symptoms, 94.2 men/95.2 women for ADL, 85.1 men/86.4 women for sports, and 85.3 men/83.6 women for quality of life. At the final follow-up evaluation, our patients had a mean age of 33.6 years and, if the two failed implantations are excluded, the average KOOS scores were 95.1 men/86.6 women for pain, 93.3 men/80 women for symptoms, 97.8 men/98.8 women for ADL, 88.7 men/69 women for sport, and 89.1 men/80 women for quality of life. The KOOS results obtained in our study are comparable with the results of Paradowski et al. [19]. The increases observed in all clinical and functional scores at the last follow-up might be related to the young age of the study population at the time of ACI. Indeed, the mean age of the participating patients was 21.33 years (range 13–45), 10 years less than the average age of patients in other studies [10–12].

With only 2 failures (13.3 %), the results of the present study compare favorably with other such reports in the literature [2, 9, 11]. Our treatment failure rate was rather low compared with the reported rates of 16–24 % observed in comparable treatment settings [8, 17]. The two failures occurred in our study due to early deterioration of the graft site; this complication always leads to clinical failure and a new intervention always becomes necessary. In both cases, it became necessary to remove the graft and treat the osteochondral damage with microfractures [20]. Four of our patients (26.6 %) showed hypertrophy of the graft on MRI at first radiologic follow-up, but in none of those cases was it symptomatic, so we did not perform a second-look arthroscopy in any of these cases. Transplant hypertrophy is a complication associated with the use of periosteum [20–23]. Thus, several modifications of the initial technique, such as periosteal flap peeling or flap substitution with synthetic membranes or fibrin matrix, were proposed to minimize its incidence and attain satisfactory results [21, 23].

In addition to providing data on long-term clinical outcomes, our study also contributes information on MRI assessment. MRI is a noninvasive method for assessing structural repair outcomes, and is considered the most effective tool for evaluating the internal structures of the knee joint. A second look via arthroscopy would enable better evaluation of the obtained repair tissue, but the invasive nature of this procedure would not allow it to be performed daily in a clinical setting. Moreover, the risks associated with such an invasive approach are not acceptable for ethical reasons, in particular for patients with satisfactory outcomes. It may only be justifiable in cases of failed treatment when the patient needs further cartilage treatment. In our study, second-look arthroscopy was only performed in two patients for whom ACI failed.

The cartilage and ACI graft were assessed with 3D sequences, which provided superior spatial resolution, aiding definition of the defect filling, the integration of the graft with the underlying bone and adjacent native cartilage, and the status of the subchondral bone and bone marrow. To describe the repair tissue, we used the previously published MOCART classification [8]. We used the MOCART score to evaluate the results after a mean follow-up period of 148.1 months. We compared the MRI findings with the clinical outcomes. Initially, the MRI variables were correlated with the subjective patient evaluation using the KOOS and IKDC scoring systems. Statistically significant correlations between the clinical outcome and some of the radiological variables were found. A statistically significant correlation of filling of the defect with KOOS pain was observed (*P* < 0.05). Effusion was statistically significantly correlated with KOOS pain and symptoms (*P* < 0.05). No statistically significant correlation was found for the other variables.

Marlovits et al. compared clinical scores with MRI variables and found statistically significant correlations with only four of the nine MOCART variables (filling of the defect, structure of the repair tissue, subchondral bone, signal intensity of the repair tissue) [5]. Our study evaluated MRI images at long-term follow-up for only ten knees and compared these data with images taken at the first follow-up. Among the nine variables of the MOCART scoring system, only one remained unchanged over time: the surface of the repair tissue. Patients who had complete or hypertrophic filling at the first follow-up presented a stable degree of defect repair over time, whilst the others who had incomplete filling showed a deterioration in the score for this variable at last follow-up (Table 3). The observed reductions in MOCART variable scores can be explained by graft aging and the alteration of the whole joint. The MOCART variables that were more likely to show reduced scores were those linked to the underlying bone alteration and to the presence of adhesions. The health and integration of the patch were seen to be compromised in a few cases. Those variations are probably linked to inflammation, which can be present in a joint that does not work optimally; moreover, the new cartilage is probably less strong than that surrounding it. Perhaps a longer follow-up period, although difficult to implement, may reveal if the MOCART score plateaus after decreasing or if it continuously decreases.

 In conclusion, first-generation ACI seems to be an effective and durable treatment for large, full-thickness chondral and osteochondral defects of the knee. ACI provides satisfactory results in terms of both pain relief and knee function rehabilitation, which appear to be sustained in the majority of patients according to long-term follow-up results. Magnetic resonance imaging plays an important role during the post-procedure follow-up of cartilage repair procedures, as it permits accurate and noninvasive assessment of the status of cartilage repair, even though there is no significant linear correlation between the overall MRI score and the subjective and objective knee scores.

Some limitations of our study need to be acknowledged. Treatment effects may have been overestimated or underestimated because of the lack of a control group. Comparison to a control group would aid accurate interpretation, as it would allow the spontaneous evolution of untreated lesions of a similar size to be evaluated. The literature provides only very limited data on patients with untreated cartilage lesions [24]. Cicuttini et al. suggested that full-thickness cartilage lesions in young patients may provoke early osteoarthritis over time [25]. To obtain more reliable data, a second study arm of patients with healthy knees or untreated chondral lesions would be of special interest. However, it should be remembered that such a control group would be difficult to create due to ethical considerations, and this remains a limitation of our analysis and other analyses of the long-term outcomes of ACI and other treatment options [2, 8, 7].
